# Different abilities of CTL specific for two HLA-A*24:02-restricted overlapping optimal epitopes to select same HIV-1 escape mutant virus

**DOI:** 10.1186/1742-4690-9-S2-P268

**Published:** 2012-09-13

**Authors:** X Sun, M Fujiwara, N Kuse, S Oka, M Takiguchi

**Affiliations:** 1 Center for AIDS Research, Kumamoto University, Kumamoto, Japan; 2 AIDS Clinical Center, National Center for Global Health and Medicine, Tokyo, Japan

## Background

Cytotoxic T lymphocytes (CTLs) are thought to exert immunologic selection pressure of escape mutation. Previous reports have showen that some overlapping peptide epitopes were presented by same HLA molecules. However, the abilities and properties of those CTLs to selection of same escape mutation are not well studied.

## Methods

CTL clones were established by stimulation of PBMC with a synthetic peptide (Nef138-8: RYPLTFGW or Nef138-10: RYPLTFGWCF) by limiting dilution method. Cytotoxic activity toward peptide-loaded cells was performed by 51Cr releasing assay. CTL suppression ability was tested by HIV-1 replication assay toward primary CD4+ cell. In vitro selection of escape mutation was performed by competitive HIV-1 replication assay. The frequency of tetramer positive cells in PBMC of HLA-A*24:02+ patients was detected by flow cytometry analysis.

## Results

Both 8-mer and 10-mer epitopes specific CTLs were established from PBMC of the patients. The ability of Nef138-10-specific CTLs to suppression HIV-1 replication in vitro was much higher than that of Nef138-8-specific CTLs. In addition, at the early stage of infection, Nef138-10-specific CTLs was predominantly elicited in the patients more than the latter ones. Cross-reactive Nef138-8-specific CTLs recognizing both WT and 2F epitopes were detected in some patients. Moreover, in vitro competitive HIV-1 assay showed that both CTLs can select escape mutants, though the ability of Nef138-10-specific CTLs was stronger than that of Nef138-8-specific ones.

## Conclusion

The present study demonstrated that Nef138-10-specific CTLs play a major role in the selection of the escape mutation, and that Nef138-8-specific CTLs also have ability to select it. We showed selection of the same escape mutants by CTL specific for same HLA-restricted overlapping epitopes.

